# Advanced image guidance for prostatic artery embolization – a multicenter technical note

**DOI:** 10.1186/s42155-021-00249-z

**Published:** 2021-08-10

**Authors:** Francisco Cesar Carnevale, Timothy McClure, Farah Cadour, Vincent Vidal, André Moreira de Assis, Airton Mota Moreira, Arthur Diego Dias Rocha, Aya Rebet, Charles Nutting

**Affiliations:** 1 Department of Radiology, University of Sao Paulo Medical School, Av. Dr. Enéas Carvalho de Aguiar, 255, São Paulo, SP 05403-000 Brazil; 2 Weill Cornell Medicine, New York, NY USA; 3 La Timone Hospital, Marseille, France; 4 GE Medical Systems, Buc, France; 5 Endovascular Consultants of Colorado Lone Tree, Lone Tree, Colorado USA

**Keywords:** Prostatic artery embolization, Benign prostatic hyperplasia, Lower urinary tract symptoms, Arterial vascular anatomy, Cone-beam computed tomography

## Abstract

**Background:**

Prostatic artery embolization (PAE) is associated with patients’ quality of life improvements and limited side effects compared to surgery. However, this procedure remains technically challenging due to complex vasculature, anatomical variations and small arteries, inducing long procedure times and high radiation exposure levels both to patients and medical staff. Moreover, the risk of non-target embolization can lead to relevant complications. In this context, advanced imaging can constitute a solid ally to address these challenges and deliver good clinical outcomes at acceptable radiation levels.

**Main text:**

This technical note aims to share the consolidated experience of four institutions detailing their optimized workflow using advanced image guidance, discussing variants, and sharing their best practices to reach a consensus standardized imaging workflow for PAE procedure, as well as pre and post-operative imaging.

**Conclusions:**

This technical note puts forth a consensus optimized imaging workflow and best practices, with the hope of helping drive adoption of the procedure, deliver good clinical outcomes, and minimize radiation dose levels and contrast media injections while making PAE procedures shorter and safer.

## Background

With transurethral resection of the prostate (TURP) being associated with higher risks of major adverse events such as bleeding, urinary incontinence, retrograde ejaculation and impotence, prostatic artery embolization (PAE) has become an important alternative for patients with lower urinary tract symptoms (LUTS) due to benign prostatic hyperplasia (BPH) who have failed pharmacotherapy (Rassweiler et al. 2006; Carnevale et al. 2013; Gao et al. 2014; Carnevale et al. 2016; Lecumberri et al. 2018; Ray et al. 2018; Hacking et al. 2019; Carnevale et al. 2020). Its benefits for patients and cost effectiveness have been demonstrated (Bagla et al. 2017; Brown et al. 2019).

One of the major technical challenges in performing PAE is the identification of and navigation through the pelvic and prostatic vascular anatomy, which presents significant variability among individuals and between pelvic sides of the same patient (de Assis et al. 2015; Carnevale et al. 2017; Bilhim et al. 2011a). In particular, prostatic arteries have highly variable origins and are frequently small, tortuous and stenotic, especially in the elderly population (de Assis et al. 2015). Although rare (Carnevale et al. 2020), non-target embolization can lead to significant complications such as rectal and bladder ischemia or penile ulcers. This challenging procedure therefore requires both excellent vascular anatomy understanding with microcatheterization skills and high precision in selecting the injection points to deliver the embolic material without reflux nor through arterial-arterial shunts (de Assis et al. 2015).

Although the use of cone-beam computed tomography (CBCT) has recently been encouraged to better understand the pelvic vascular anatomy (Bagla et al. 2013; Wang et al. 2017; Rocha et al. 2020; Cadour et al. 2020; Bagla and Sterling 2014; Schnapauff et al. 2020), PAE remains technically challenging, typically resulting in long procedure and fluoroscopy times, and high radiation dose due to the need for multiple oblique digital subtraction angiographies (DSA) and magnified views, with significant variability between centers (median DAP ranging from 33.2 to 863.4 Gy∙cm2 between individual studies) (Laborda et al. 2015; Garzon et al. 2016; Andrade et al. 2017; Maclean et al. 2017; Tanaka et al. 2017; Zumstein et al. 2020). In this context, a refined technique is crucial for technical and clinical success. If the embolization technique has been well described (Cornelis et al. 2020; Carnevale et al. 2014), little has been said about image guidance available to the Interventional Radiologist.

This technical note aims to share the consolidated experience of physicians from four institutions performing a total of 300 PAEs per year, on average. A consensus imaging workflow, optimized for PAE, is described, some variants discussed, and best practices using advanced image guidance shared, in hopes of providing the Interventional Radiology community with a resource to deliver good PAE technical and clinical outcomes at acceptable radiation dose levels.

This retrospective analysis was approved by local institutional review boards and exempted from patient informed consent.

## Consensus imaging workflow

### Methods for reaching consensus

**Fig. 1:**
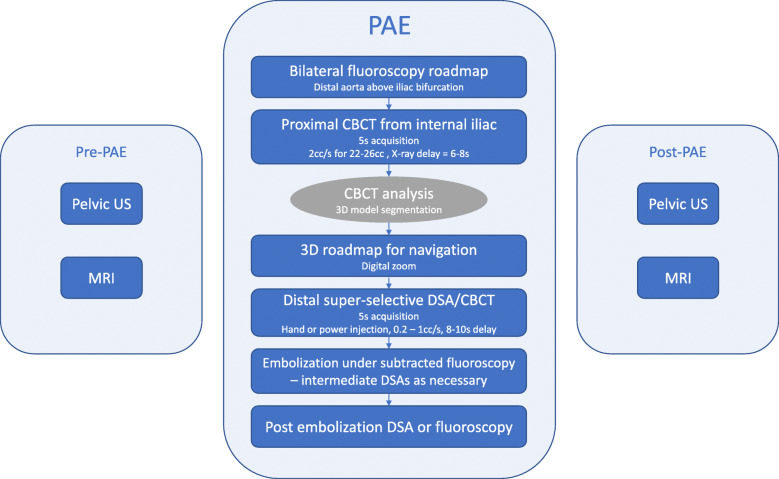
Imaging workflow summary. Detailed steps of the proposed pre, post and intraprocedural PAE imaging workflow for each side, from planning to guidance and assessment

Six PAE users from four institutions, with 4 to 13 years of experience in PAE, gathered to discuss their specific techniques and propose a consensus imaging workflow. Reviews were organized for each user to present their imaging workflow and technique, allowing to highlight commonalities and differences and to share best practices, which were then prospectively tested by the other authors to feed the following discussions, resulting in an optimized imaging workflow and technique adopted by all centers (Fig. 1). This workflow was established with the objective of better visualizing the anatomy, better planning, guiding and assessing the procedure, while minimizing radiation exposure to patients and clinical staff.

### Pre-operative imaging

Understanding the specific patient pelvic anatomy is an important step of the procedure planning phase (de Assis et al. 2015). Pre-procedural computed tomography (CT) and magnetic resonance (MR) angiography can be useful to understand the main prostatic artery supply (Bilhim et al. 2011b; Kim et al. 2018). However, the non-selective injection and CT’s and MR’s limited spatial resolution make the identification of small prostatic arteries challenging (Maclean et al. 2018), with intraoperative CBCT shown to allow for better prostatic artery identification with improved signal-to-noise and contrast-to-noise ratio and with less radiation dose compared to conventional CT (Desai et al. 2018). In all centers experience, CT and MR’s spatial resolution was too limited for detailed anatomical analysis (e.g. when trying to localize prostatic artery origins and distal connections, anatomy pathway, and anastomoses with neighboring structures). With superior contrast resolution, pre-operative MR is still recommended to confirm BPH, rule out prostate cancer, estimate zonal and total prostatic volumes and plan for PAE treatment, especially for smaller prostates (Guneyli et al. 2016; de Assis et al. 2017). Reasons to use pre-PAE CTA would include understanding of the pelvic and main prostatic vascular anatomy, selection of arterial access (femoral or radial) and selection of catheters to be used during the procedure. However, intra-procedural CBCT is the best imaging tool to understand the prostatic arteries, intraprostatic anastomosis and the prostate zone that each prostatic branch is feeding. Pre-operative CT is not recommended to minimize overall patient radiation exposure and contrast. Pelvic US is recommended to measure the postvoid residual volume.

### Intraprocedural imaging - general guidelines

During PAE procedure, DSA offers excellent spatial resolution from selective injection points; however, the projective nature of DSA images can be misleading when analyzing small and tortuous prostatic arteries, prompting the needs for multiple projections, which typically results in increased levels of radiation exposure, contrast medium and procedure time. CBCT has been increasingly adopted and recommended during IR procedures, allowing a precise assessment of complex vascular anatomy in 3D with a single injection of contrast medium in a selectively targeted artery. Its benefits include high spatial resolution combined with an intra-arterial injection of a smaller volume of contrast compared to CT. Its value during PAE compared to preoperative CT and to DSA has already been demonstrated (Bagla et al. 2013; Wang et al. 2017; Rocha et al. 2020; Cadour et al. 2020; Bagla and Sterling 2014; Schnapauff et al. 2020; Desai et al. 2018). Compared to DSA, CBCT provided information that impacted treatment in 46% of PAE patients by allowing identification of potential sites of non-target embolization (Bagla et al. 2013). Recent studies concluded that the number of prostatic arteries origins and anastomoses that could be identified were significantly higher with CBCT than with DSA (Wang et al. 2017), and that CBCT provided essential information that was not available with DSA in 46% (Rocha et al. 2020) to 60.8% (Wang et al. 2017) of patients. Finally, advanced imaging techniques such as 3D road-mapping should be utilized as they can contribute to both performing safer and more efficient procedures, and reducing x-ray dose and procedure time (Hertault et al. 2018; Schott et al. 2019).

### Intraprocedural imaging detailed workflow

To provide a complete overview of the pelvic anatomy, an initial bilateral non-selective fluoroscopic roadmap is recommended from the distal aorta just above the aortic common iliac bifurcation. This roadmap is helpful in understanding internal iliac arteries origin as well as in providing the length of the common and internal iliac arteries to support catheter selection (Laborda et al. 2015). In this group’s experience, hand-injected fluoroscopic roadmap of 50% diluted contrast provides sufficient image quality at this stage, and decreases radiation exposure compared to DSA.

**Fig. 2:**
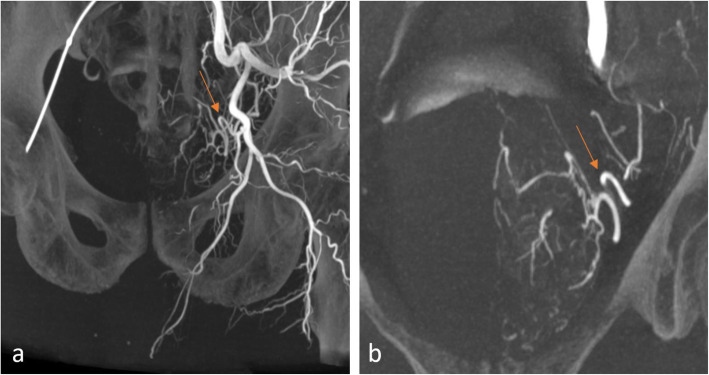
Proximal CBCT. Five seconds CBCT acquisition, injection from left internal iliac artery (Discovery IGS 740, GE Healthcare, Chicago, IL). Maximum Intensity Projection (MIP) coronal views showing the left pelvic arterial vascular anatomy (**a**) and the left prostatic artery (arrow) and prostate gland blush (**b**)

PAE usually consists of a bilateral successive embolization, often starting from the left side because of the designed use of the Robert’s uterine and the Carnevale’s prostate catheters when performed through the femoral approach. For each side, a first proximal 5 s CBCT is acquired with the 5 French catheter in the internal iliac artery, above the bifurcation of the anterior and posterior branches (Carnevale et al. 2014). The following injection parameters are typically used: 22 to 26 cc of pure contrast, injected at 2 cc/s, with an X-ray delay of 6 to 8 s. This injection protocol ensures a good filling of the arteries during the entire spin, thus allowing an adequate visualization of both the arterial anatomy and the prostate parenchymal blush in a single CBCT (Fig. 2).

With the 180 degrees rotational DSA-like image provided by the CBCT spin, and with recent studies showing CBCT’s superiority to DSA for PAE planning (Wang et al. 2017; Rocha et al. 2020), it is recommended to skip the typically acquired ipsilateral oblique DSA from the internal iliac artery (Carnevale and Antunes 2013) to limit patient radiation exposure, contrast medium use and reducing the procedure time. Another variant to further reduce radiation dose consists of acquiring the proximal CBCT bilaterally from the distal aorta, with 60 cc of contrast injected at 6 cc/s with an X-ray delay of 6 s. In the group’s experience, this CBCT does not provide as much distal information of the prostatic arteries. Based on this experience, and given the lower prevalence of type V cases (de Assis et al. 2015), the recommendation is to start straightforwardly with the internal iliac artery CBCT. It provides a rich source of anatomical information with the adequate level of distality and selectivity in most cases. In practice, CBCTs can be acquired arms down or with arms on the chest with limited impact on image quality thanks to automatic exposure optimization (IGS5/740, GE Healthcare).

**Fig. 3:**
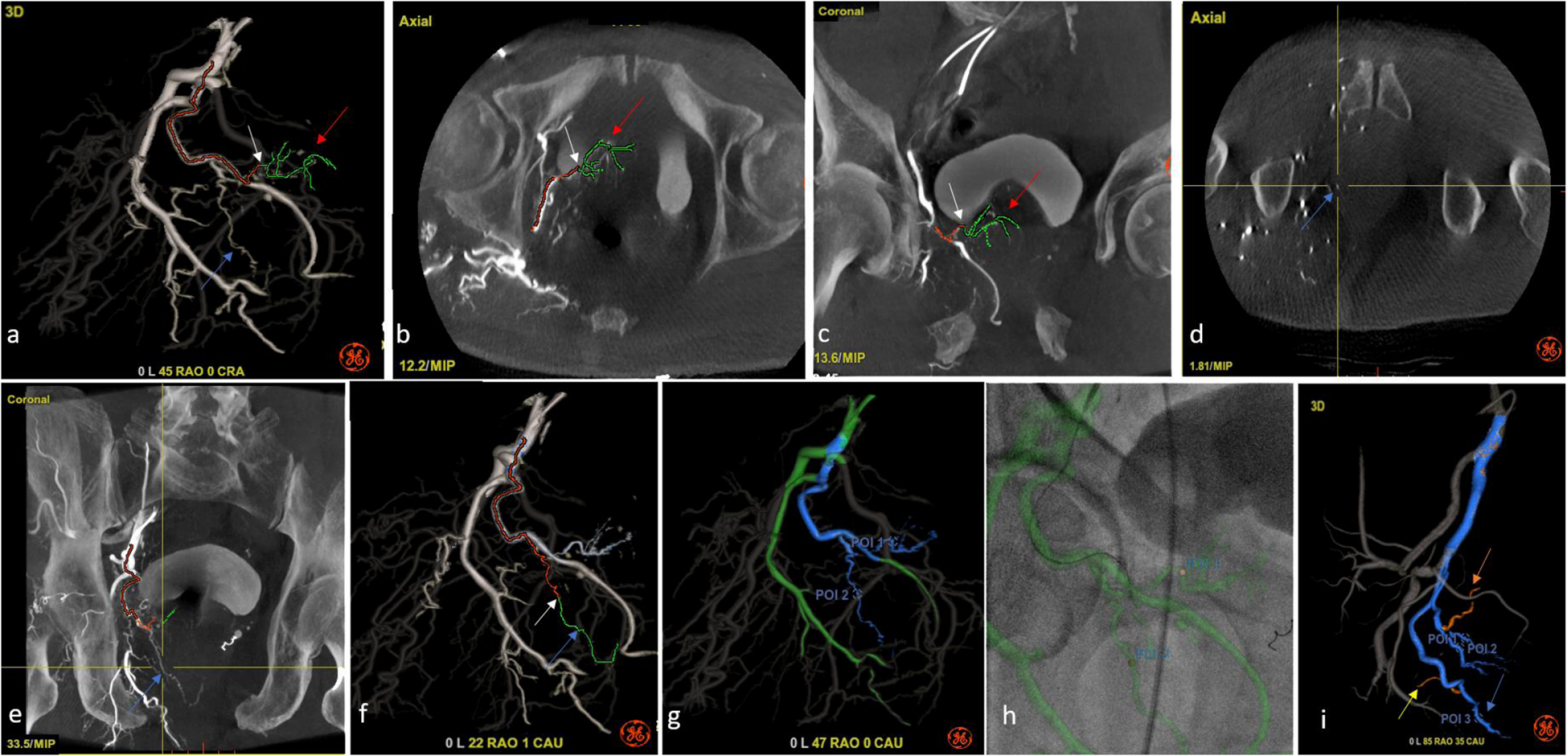
Advanced planning on proximal CBCT using Virtual Injection technology (Embo ASSIST, GE Healthcare). Automatic arterial segmentation and bone removal, with Virtual Injection used to highlight the main prostatic artery (red arrow) (**a**-**c**). Distality from, and path to the virtual injection point (white arrow) are indicated in green and red, respectively. A separate secondary prostatic artery (blue arrow) was thus detected since unperfused by the injection simulation (**d**-**f**). This branch was initially taken to be a rectal branch on the 3D volume rendering (**a**), but confirmed as a prostatic artery with Virtual Injection (**f**). Both prostatic arteries were saved as 3D guidance model (**g**) to facilitate their catheterization using augmented fluoroscopic guidance (**h**). Prostatic arteries are indicated in blue, with planned points of injection (POI) marked on the overall navigation 3D model (in green). Separate case (**i**) showing how advanced planning with Virtual Injection allowed detection of a bladder branch (orange arrow) arising from the central prostatic artery, inducing the need for two separate distal points of injection (POIs 1 & 2), as well as a capsular branch (blue arrow), with POI3 defined distally from a branch going to the rectum (yellow arrow). 3D model for augmented fluoroscopic guidance highlighting path to prostatic arteries (in blue), 3 planned points of embolization, and non-target branches to avoid (in orange)

Proximal CBCT datasets should be analyzed carefully to identify arteries feeding the prostate, their pathways and non-target vessels. Prostatic arteries should be identified exhaustively and bilaterally to maximize treatment completion and reduce symptom recurrence risks (de Assis et al. 2019a). Advanced planning software such as Virtual Injection (Embo ASSIST, GE Healthcare) allows to simulate selective injections based on a proximal CBCT, facilitating the identification of prostatic arteries and non-target vessels (Fig. 3). Automatic segmentation of the pelvic and prostatic vasculature and of the arteries of interest, both prostate feeders and non-target vessels, along with their centerlines, facilitates procedure planning from table side and creates a 3D model for augmented fluoroscopy (Fig. 3).

During microcatheter navigation (≤ 2.4 French recommended), this 3D model is overlaid on the live fluoroscopy, providing a 3D roadmap that is and remains automatically registered to the patient despite gantry and table movements. In case of patient motion, the 3D roadmap registration can be adjusted from table side to better match the patient. Depending on the procedure stage, both the prostatic artery to select and the non-target arteries to avoid can be interesting to display on fluoroscopy. In addition to its benefits facilitating catheter guidance, the availability of the 3D roadmap allows to select the best working angulations to visualize vessel turns and bifurcations without using fluoroscopy thus with no radiation. The 45 degree ipsilateral angle typically recommended should be replaced by a systemic identification of the optimal working angle based on patient’s anatomy using 3D roadmap without fluoroscopy, which may be less steep than 45 degrees and thus more radiation effective. Multiple DSA runs can thus be avoided by using the CBCT-based 3D roadmap, resulting in both dose and contrast savings.

During microcatheter navigation, basic ALARA radiation best practices should also be enforced to minimize radiation exposure both to patients and medical staff: use of digital zoom at maximum FOV instead of magnification; rigorous collimation; low default dose settings and frame rates, e.g. 1 fps for DSA and 3.75 fps for fluoro, which can be increased when needed from table side; fluoroscopy storing instead of DSA when sufficient in terms of image quality; and avoiding steep angulations when possible to minimize scatter radiation and optimize image quality.

**Fig. 4:**
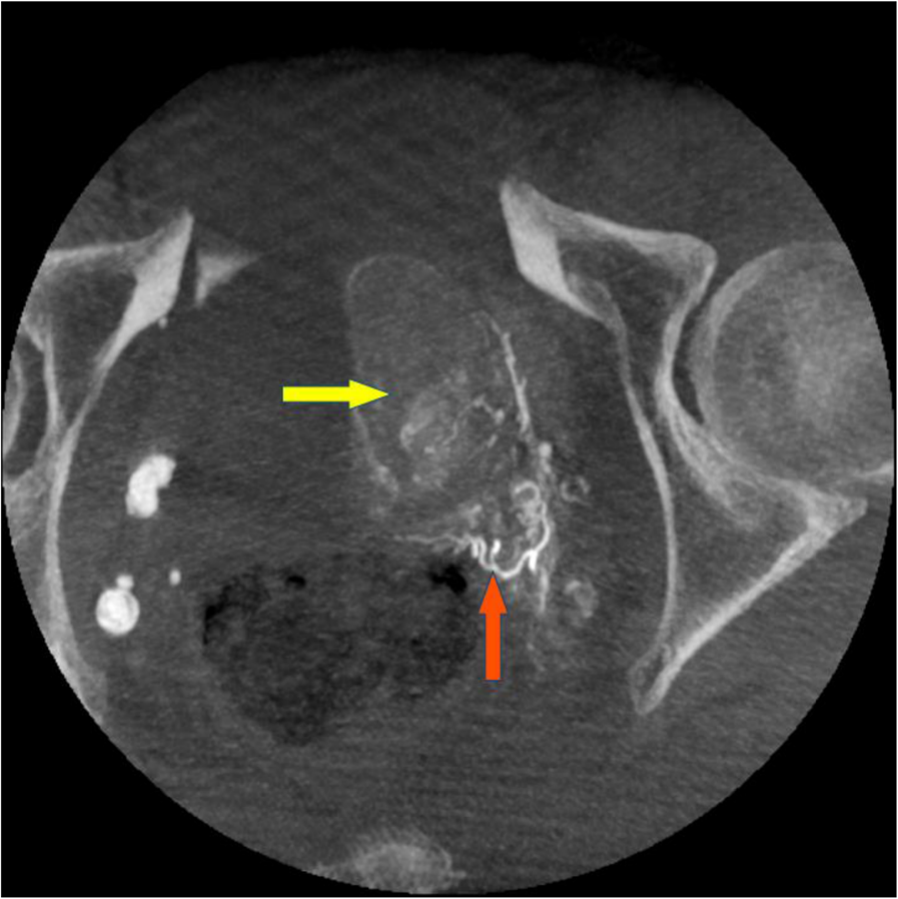
Distal CBCT. Ten millimeters MIP axial view from a super-selective CBCT showing prostatic blush (yellow arrow) as well as rectal branch (orange arrow) originating from a prostatic artery, observed after vasodilator injection. The catheter had to be repositioned to avoid off-target embolization

Once the microcatheter is in the desired location within the prostatic artery, a distal DSA in ipsilateral oblique view with 3-5 cc of 50–70% contrast hand-injected or power-injected at 0.2 to 1 cc/s (depending on the prostate size, prostatic artery diameter and collaterals) is recommended to confirm no non-target vessels are being perfused, as well as to simulate the embolization treatment by visualizing the prostate perfusion. When further confirmation is needed, e.g. to clearly distinguish prostatic from rectal branches, or if both central gland and peripheral zone arterial branches are not observed, or for early experience PAE users, a distal 5 s CBCT is recommended with either hand or power injection with an 8–10 s delay to ensure good filling of both the prostate and any extra-prostatic structure (Fig. 4). In this group’s experience, hand injection gives better control to fill the prostate, avoid reflux and obtain a strong injection to identify shunts. For hand injected CBCTs, half of the contrast is injected before the spin starts to optimize contrast uptake in the prostate and surrounding organs; the other half is injected during the spin rotation to confirm the local arterial anatomy and help evaluate risks of reflux in non target anatomies. Operators should not be present in the examination room during CBCT acquisition. If required due to manual injection preferrence, operators should stand behind lead shields to minimize their own radiation exposure. To avoid operator presence in examination room, power injection with PSI at 300 can help avoid reflux during CBCT acquisition.

Intra-arterial vasodilator should be used when navigating stenotic and/or tortuous anatomies, before DSA/CBCT acquisition and before embolic agents injection, with the aim of opening the prostatic vascular bed. Isosorbide mononitrate (5-10 mg) and nitroglycerin (100 μg) are typically used. Prostate enhancement can be better observed after vasodilator injection. Some shunts, mostly common to the internal pudendal artery, rectum and bladder, can also be more evident after vasodilator injection.

Once distal DSA/CBCT analysis confirms the microcatheter location for treatment, the embolic material is delivered proximally first, then deeper in the gland, following the PErFecTED technique (Carnevale et al. 2020; Zumstein et al. 2020). Postero-anterior incidence can be used for types I, II and III with the aim of reducing radiation exposure because they are longer and there is less risk of embolic agent reflux in these types of arteries. Ipsilateral oblique view is recommended for type IV because of the risk of proximal embolic agent reflux to the internal pudendal artery. Microspheres ranging from 100 to 500 μm have been used. Adverse events and complications seem to be more frequent with smaller particles due to deeper penetration and passing through anastomosis (Carnevale et al. 2020; Gonçalves et al. 2016). During this time-consuming step of embolic agent injection, low dose fluoroscopy at 3.75fps can be used to monitor the embolization, in unsubtracted or subtracted mode, and stored for documentation purposes. Intermittent 0.5fps DSAs can be used as necessary. Collimation, digital zoom instead of magnification, and use of postero-anterior views are particularly recommended to reduce radiation in this step. A final 0.5fps DSA or stored fluoroscopy allows to control the success of the embolization, defined as total stasis. The catheter is then navigated to the other side where the workflow is repeated.

### Post-procedural imaging

For post-PAE it’s recommended pelvic US to measure the postvoid residual volume and prostate size. MRI should be used to a better understanding of the ischemic areas and prostate reduction, mainly in patients with poor results or for research’s aims. The recommendation is 3 months after the procedure and then an annual follow up.

## Conclusion

Prostatic artery embolization has demonstrated to deliver quality of life and symptoms improvements, reducing the size and consistency of the prostate (de Assis et al. 2019b) with limited side effects compared to surgery. However, little has been published on optimal imaging to guide this technically challenging procedure.

This technical note puts forth a consensus optimized imaging workflow and best practices, with the hope of helping drive adoption of the procedure, deliver good clinical outcomes, and minimize radiation dose levels and contrast media injections while making PAE procedures shorter and safer. This assumption will be addressed in future studies.

## Data Availability

Not applicable.

## Abbreviations

TURP: Transurethral resection of the prostate
PAE: Prostatic artery embolization
BPH: Benign prostatic hyperplasia
CBCT: Cone-beam computed tomography
DSA: Digital subtraction angiography
LUTS: Lower urinary tract symptoms
CT: Computed tomography
MR: Magnetic resonance
